# Gene Network of Susceptibility to Atypical Femoral Fractures Related to Bisphosphonate Treatment

**DOI:** 10.3390/genes13010146

**Published:** 2022-01-14

**Authors:** Natalia Garcia-Giralt, Neus Roca-Ayats, Josep F Abril, Nuria Martinez-Gil, Diana Ovejero, Santos Castañeda, Xavier Nogues, Daniel Grinberg, Susanna Balcells, Raquel Rabionet

**Affiliations:** 1 Musculoskeletal Research Group, IMIM (Hospital del Mar Medical Research Institute), Centro de Investigación Biomédica en Red en Fragilidad y Envejecimiento Saludable (CIBERFES), ISCIII, 08003 Barcelona, Spain; dovejero@imim.es (D.O.); xnogues@parcdesalutmar.cat (X.N.); 2 Department of Genetics, Microbiology and Statistics, Faculty of Biology, Universitat de Barcelona, CIBERER, IBUB, IRSJD, 08028 Barcelona, Spain; NEROCA@clinic.cat (N.R.-A.); jabril@ub.edu (J.F.A.); airun91@gmail.com (N.M.-G.); dgrinberg@ub.edu (D.G.); sbalcells@ub.edu (S.B.); kelly.rabionet@ub.edu (R.R.); 3 Department of Rheumatology, Hospital Universitario de La Princesa, IIS-Princesa, Cátedra UAM-Roche, EPID-Future, Universidad Autónoma de Madrid, 28670 Madrid, Spain; scastas@gmail.com

**Keywords:** atypical femoral fractures, bisphosphonates, WES

## Abstract

Atypical femoral fractures (AFF) are rare fragility fractures in the subtrocantheric or diaphysis femoral region associated with long-term bisphosphonate (BP) treatment. The etiology of AFF is still unclear even though a genetic basis is suggested. We performed whole exome sequencing (WES) analysis of 12 patients receiving BPs for at least 5 years who sustained AFFs and 4 controls, also long-term treated with BPs but without any fracture. After filtration and prioritization of rare variants predicted to be damaging and present in genes shared among at least two patients, a total of 272 variants in 132 genes were identified. Twelve of these genes were known to be involved in bone metabolism and/or AFF, highlighting *DAAM2* and *LRP5*, both involved in the Wnt pathway, as the most representative. Afterwards, we intersected all mutated genes with a list of 34 genes obtained from a previous study of three sisters with BP-related AFF, identifying nine genes. One of these (*MEX3D*) harbored damaging variants in two AFF patients from the present study and one shared among the three sisters. Gene interaction analysis using the AFFNET web suggested a complex network among bone-related genes as well as with other mutated genes. BinGO biological function analysis highlighted cytoskeleton and cilium organization. In conclusion, several genes and their interactions could provide genetic susceptibility to AFF, that along with BPs treatment and in some cases with glucocorticoids may trigger this so feared complication.

## 1. Introduction

Atypical femoral fractures (AFF) are a very rare type of bone fractures associated mainly with bisphosphonates (BP) and very rarely also with denosumab use [1,2,3]. Genetic factors have been suggested as a possible explanation for both the higher risk of AFF in Asian populations and the low proportion of BP users that develop AFF [4].

Many attempts have been made to identify these genetic factors that may predispose some BP users to sustain AFF. Among them, a few studies have revealed that genetic variants in genes implicated in the mevalonate pathway, which is targeted by BP, may affect bone mineral density, bone turnover, and predispose to AFF, in response to BP treatment [5,6,7,8]. However, a recent genome-wide association study (GWAS) and candidate gene study comparing 51 AFF cases to 324 BP-treated controls was unable to find evidence of common genetic variants for BP-associated AFF [9]. Hence, the authors proposed to perform GWAS with a larger sample size as well as whole-exome or whole-genome sequencing studies. This combination of studies would help to uncover the genetic background associated with BP-related AFF, which has a high genetic heterogeneity, sometimes associated with monogenic disorders [10,11] or otherwise with a polygenic etiology and large variability among individuals [12,13].

Here, we performed whole exome sequencing of 12 patients with BP-associated AFF and 4 BP-treated controls to identify genes involved in AFF susceptibility.

## 2. Materials and Methods

### 2.1. Subjects

Twelve unrelated postmenopausal women with AFF and four postmenopausal women without any fracture (controls) were recruited, all of them having received long-term (>5 years) BP treatment due to a diagnosis of osteoporosis. All women were Caucasian recruited in Hospital del Mar (Barcelona, Spain) and Hospital Universitario de La Princesa (Madrid, Spain). In order to establish the diagnosis of AFF, we used the revised criteria of the American Bone and Mineral Research Taskforce [14]. Baseline characteristics of AFF patients and controls are described in Table 1. No patient had hypophosphatemia or any diagnosed monogenic disease. Half of the AFF patients received corticosteroid therapy due to polymyositis, rheumatoid arthritis, asthma or chronic bronchitis.

### 2.2. Whole-Exome Sequencing (WES)

DNA of participant subjects was extracted from peripheral blood with the Wizard Genomic DNA Purification Kit (Promega, Madrid, Spain) and sequenced at the Centro Nacional de Análisis Genómico (CNAG) facilities (Barcelona, Spain). Capture was performed using Agilent Human All Exon 50 Mb v5 and samples were sequenced at a coverage of 140× on a HiSeq 2000 sequencer. Basic bioinformatic processing of the sequencing data was performed using the CNAG’s in-house pipeline [12]. Genetic variants were filtered according to the following premises: (1) Coverage (DP) ≥ 10; (2) Genotype Quality ≥ 30; (3) exclusion of synonymous variants; (4) Minor Allele Frequency (MAF) in ExAC and CSVS (Collaborative Spanish Variability Server: http://csvs.babelomics.org/ accessed on 9 December 2021) ≤ 0.005; (5) absent in BP-treated controls. Finally, SIFT [15], PolyPhen [16], and CADD [17] prediction tools were used for prioritization.

### 2.3. AFF Network Construction (AFFNET)

High-throughput interaction data were retrieved from BioGRID (version 3.4.133) [18,19] and STRING [Search Tool for the Retrieval of Interacting Genes/Proteins, version 10 [20]], with additional information from GeneOntology (http://geneontology.org, accessed on 9 December 2021), GeneCards (https://www.genecards.org/, accessed on 9 December 2021), OMIM (https://www.omim.org/, accessed on 9 December 2021), UniProt (https://www.uniprot.org/, accessed on 9 December 2021), RefSeq (NCBI), and gnomAD (Genome Aggregation Database: https://gnomad.broadinstitute.org/, accessed on 9 December 2021). This whole human gene/protein interaction network included 26,934 nodes and 794,052 edges.

A Perl script was implemented to capture the interactions subnetwork using AFF genes to find all possible pair-wise shortest paths by applying the Dijkstra algorithm implemented in the Graph Perl module. The Graph: Directed module was used to define the whole network data structure as a directed graph, which simplified the calculations for the AFF subnetwork. The script produced a skeleton graph on top of the whole interactions graph stored into a Neo4j (https://neo4j.com/, accessed on 9 December 2021) database, to make data available on the AFFNET web interface (https://compgen.bio.ub.edu/AFFgenes/, accessed on 9 December 2021, available upon request). This web interface was developed for user-friendly network exploration by researchers. It was implemented via Django (https://www.djangoproject.com/, accessed on 9 December 2021) to process queries, integrate the data, and display the resulting network through the open-source Cytoscape JavaScript library for graph analysis and visualization [21]. The main web form provides one entry point that focuses on selected genes (similarly to other current gene/protein browsers). The web display facilitates interaction with the nodes and edges by zooming, displacing, changing the graph layout, adding or removing nodes, and retrieving information about AFF genes and their interactions. The border color of the nodes identifies them as genes mutated in a minimum of 2 AFF patients (purple), or genes related to bone metabolism (grey). The filling core of the nodes represents osteoclast gene expression, which was retrieved from the GSE database GSE63009: Osteoclastic precursor cells treated or not with bisphosphonates (alendronate or risedronate) during their differentiation into mature osteoclasts [22]. The color scale goes from red (overexpressed) to dark blue (underexpressed), with yellow indicating no change of expression. For this specific task, a standard protocol based on the Bioconductor [23] limma R package was run.

## 3. Results

### 3.1. Variant Selection

**Figure 1:**
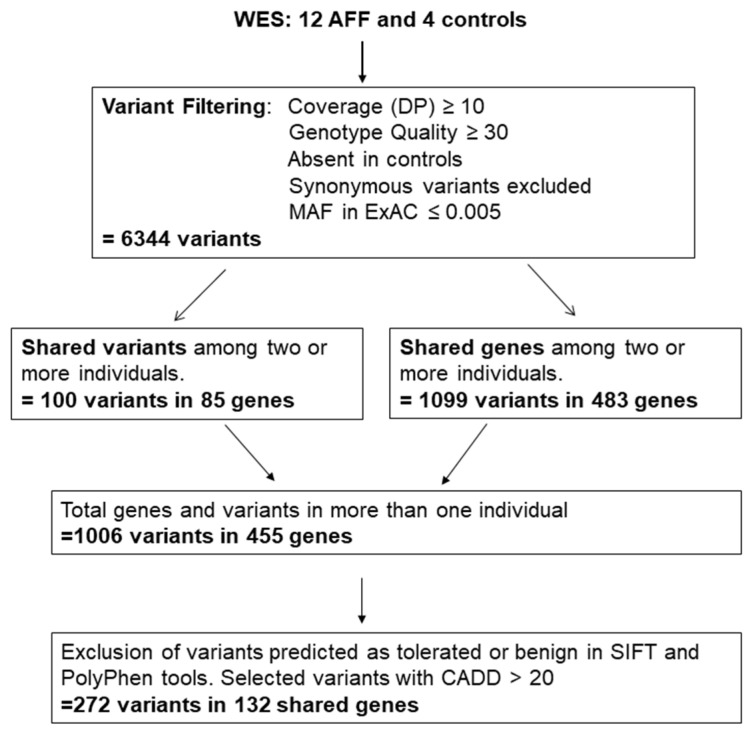
Pipeline of selected variants obtained by whole exome sequencing of 12 patients with BP-related AFF and 4 controls (individuals with long-term BP treatment without AFF). Only variants or genes mutated in at least two patients were considered for further analysis.

In order to identify genes putatively involved in AFF, we first removed all variants identified in the four control samples, and then selected those genes harboring rare genetic variants (ExAC and CSVS < 0.005) in at least two patients (Figure 1). We identified 100 rare variants in 85 genes that were shared by at least two patients. In addition, 483 genes presented a rare variant in at least 2 patients with AFF (same gene, different variant). In total, 1006 variants in 455 genes were identified (Table S1).

Variants were then prioritized based on functional prediction (excluding variants with CADD score < 20, and those considered tolerated or benign by SIFT or PolyPhen_humDiv, respectively). Considering only genes with at least two carriers of a rare variant, a total 272 variants in 132 genes remained (Figure 1 and Table 2).

Function enrichment analysis using the BinGO and GeneMANIA app in Cytoscape yielded adjusted significant scores for dynein complex, contractile fiber, microtubule motor activity, ciliary transition zone, actin cytoskeleton organization and pyrophosphatase activity (Figure S1).

Afterwards, we intersected this list with previously described genes involved in bone metabolism and/or AFF [13,24,25,26]. Twelve genes were identified and selected as candidate genes (Table 3) for further in silico analyses using the AFFNET tool. Half of the AFF patients were carriers of variants in one Wnt signaling gene: *DAAM2* (3 carriers, one each for p.(P555L) (homozygous), p.(P582H) and p.(R989L), and a fourth with a variant predicted as tolerated by SIFT (p.(K776T))) and *LRP5* (3 carriers, one each for p.(R258C) and p.(P1504L) and one carrying two variants (p.(R1036Q) and p.(S1482L)), suggesting a role of this pathway in AFF triggering.

**Figure 2:**
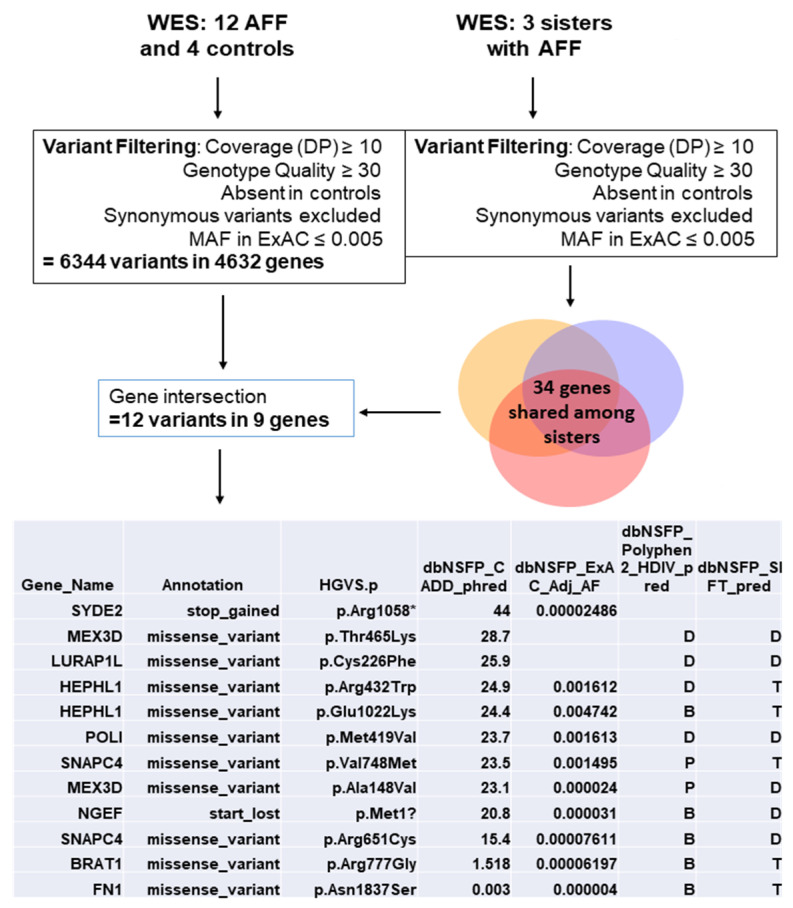
All mutated genes from the WES were intersected with genes also mutated in a previous study with 3 sisters who sustained AFF [12].

In parallel, we compared all genes carrying rare variants in this study with previous results obtained from a WES in three sisters with BP-related AFF [12]. A total of 9 genes were found overlapping both studies (Figure 2). Four of them carried damaging rare variants: *LURAP1L*, *MEX3D*, *POLI*, and *SYDE2*. These genes were also considered candidate genes for the network analysis.

### 3.2. AFF Network Analysis with Candidate Genes

**Figure 3:**
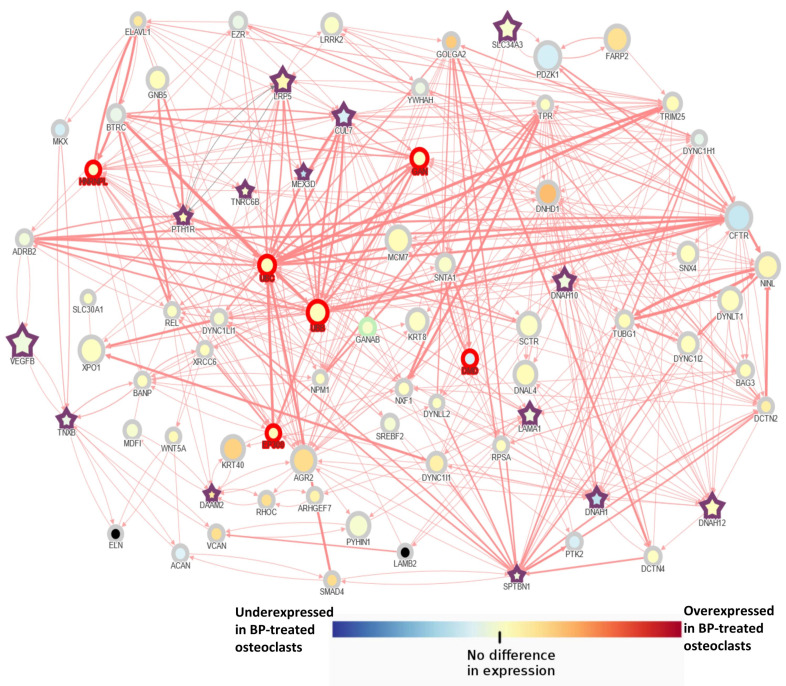
Network analysis of bone-related genes mutated in at least 2 AFF patients using the AFFNET tool. The shortest path interactions among candidate genes were displayed. Purple stars indicate candidate genes. The size of the nodes is determined by observed/expected loss of function score in the gnomAD database. This score is the ratio of the observed and expected loss of function variants in a particular gene. This score provides insight into how tolerant a gene is to loss of function variation. The red border tags genes that are outlier in the constrain metrics for LoF or missense variants according to gnomAD. Node color represents their expression depending on the expression data obtained in the GSE63009: Osteoclastic precursor cells treated or not with bisphosphonates (alendronate or risedronate) during their differentiation into mature osteoclasts.

Interactions among identified genes were explored using the AFFNET tool. In order to simplify the network display, the shortest path interactions among bone-related genes (described in Table 3) were explored. Therefore, only direct interactions between candidate genes are displayed. Candidate genes were interconnected with each other, even though in some cases through other intermediate genes (Figure 3).

Besides, a very complex network with multiple interactions among candidate genes and with the other mutated genes (Table 2) is displayed when all possible interactions were included in the analysis (Figure S2). Finally, interactions of mutated genes overlapped between the present study and the previous study with 3 sisters with AFF (Figure 2) were also explored. No direct interactions were found among these genes even though they were interconnected through one intermediate gene (Figure S3). One gene, *LURAP1L*, had no interactions with other genes. The *MEX3D* gene, which is mutated in 2 patients plus the 3 sisters, is under expressed in BP-treated osteoclasts (Figure S3).

## 4. Discussion

AFF are rare low-trauma fragility fractures in the subtrocantheric or diaphysis femoral region. They are considered a potential rare side effect of long-term BP treatment according to the task force of the American Society for Bone and Mineral Research [14]. Although AFF have been reported to be related to several monogenic diseases like hypophosphatasia (HPP), X-linked hypophosphatemia, pycnodysostosis, osteopetrosis, osteoporosis pseudoglioma syndrome (OPPG), osteogenesis imperfecta (OI), and X-linked osteoporosis, in most cases, AFF does not occur in the setting of known monogenic causes. In these cases, other rare variants in genes related to bone metabolism pathways may be involved in the AFF susceptibility.

Here, we present a WES analysis of 12 patients receiving BPs for at least 5 years, without evidence of monogenic disease association. In these patients, BPs were prescribed due to postmenopausal osteoporosis. After filtration and prioritization of rare variants predicted to be damaging and present in genes shared among at least two patients, a total of 272 variants in 132 genes were identified. Twelve of these genes were involved in bone metabolism and/or AFF, according to previous studies [12,13,24,25,26]. Two of these genes, *DAAM2* and *LRP5*, both involved in the Wnt pathway, were mutated in 3 patients each.

Finally, we intersected all mutated genes with a list of 34 genes obtained from a previous study of three sisters with BP-related AFF [12]. Nine genes were obtained, one of them (*MEX3D*) harboring damaging variants in two AFF patients (Table 2), in addition to the three sisters variant p.Thr560Arg (with a SIFT = 0.03 and CADD = 15.51). Of note, this gene was found under expressed in BP-treated osteoclasts according to the GSE63009 database. The role of this gene in bone tissue remains to be explored.

Our WES findings along with gene network analysis suggest a multigenic model in which an accumulation of susceptibility variants may lead to a predisposition to BP-related AFF. Furthermore, each individual carries its own genetic signature, making it difficult to establish a general pattern for AFF. It is noteworthy that our patients were treated with BPs due to osteoporosis and, therefore, also had a genetic background predisposing to skeletal fragility which adds more complexity to the gene network. Hence, AFF could be the result of the interaction of genes involved in osteoporosis plus specific genes involved in AFF, without disregarding the role of BP in this scenario. Furthermore, half of the patients with AFF also received corticosteroid treatment, which is involved in both osteoporosis and AFF development [28]. Glucocorticoids activate osteoclasts function and the suppression of osteoblasts as well as osteocyte apoptosis [29]. However, the degree of the involvement and the mechanisms underlying in the risk of AFF are still unknown. Denosumab treatment was administered in few patients (two AFF patients and one without AFF; not statistically significant) who received only one dose. We think that denosumab is not playing an important role in these patients although it is an antiresorptive drug also involved in the AFF susceptibility [30].

In order to build gene networks, we selected candidate genes because of their function or association with bone biology or AFF, as previously described in the literature. The most prominent genes were *DAAM2* and *LRP5* since they belong to the Wnt signaling pathway and each of them was found mutated in 3 AFF patients. There was one additional carrier of a rare *DAAM2* variant, which did not pass the prioritization threshold, as SIFT classified it as tolerated even though PolyPhen and CADD predictions classified it as potentially damaging. *LRP5* gene is a well-known bone-related gene involved in bone metabolism and monogenic bone diseases and phenotypes [31]. Of note, AFF occurred in one case of osteoporosis pseudoglioma syndrome associated with two novel compound heterozygous mutations in *LRP5* [32]. On the other hand, the role of *DAAM2* in AFF pathophysiology is supported by murine studies given that a *DAAM2* knockout mouse showed decreased bone strength, not only as a result of abnormal bone turnover, but also as a consequence of increased porosity and impaired bone composition and structure [24]. Moreover, *DAAM2* was reported to promote osteoclastic bone resorption via the *Daam2*-Rho-Pkn3-***c***-Src pathway [33]. Interestingly, three of the patients with *DAAM2* rare variants had received glucocorticoid treatment. The fourth patient, who had not been treated with corticosteroids, was homozygous for a predicted damaging variant. *DAAM2* was identified as an osteocyte signature gene and was expressed in mouse calvaria osteoblasts and bone marrow-derived osteoclasts. Functional analysis of *Daam2* in the bone context was explored in both SaOS-2 cells and mice osteoclasts suggesting an important role in bone remodeling [24,33]. However, the role of glucocorticoids in AFF susceptibility as well as its interaction with mutations in *DAAM2* remains to be explored and further studies should be performed in patients with AFF exposed to glucocorticoids.

Other interesting candidate genes included: *SLC34A3*, involved in hereditary hypophosphatemic rickets with hypercalciuria, a rare autosomal recessive disorder [34]; *CUL7*, involved in 3 M syndrome, an autosomal recessive disorder characterized by severe growth retardation, distinct facial features, and skeletal changes; *TNXB,* known to be the gene responsible for classic-like Ehlers-Danlos syndrome, in which TNX deficiency in the bone marrow promotes multinucleation of osteoclasts and results in increased bone resorption activity [35]; and *PTHR1*, which encodes for the parathyroid hormone receptor and whose defects are known to be the cause of Jansen’s metaphyseal chondrodysplasia (JMC), chondrodysplasia Blomstrand type (BOCD), as well as enchondromatosis.

It is noteworthy that these genes interacted either directly or indirectly between each other and with a number of other mutated genes, highlighting the complexity of the AFF. For example, in the case of the dyneins, DNAH10 and 12, that are microtubule-dependent motor proteins, interacted among them, both directly or through other intermediate proteins of the dynein family. Interestingly, the expression of some of these dyneins is altered in osteoclasts treated with BPs. Besides, *Daam2* interacted with LAMA1 through Rho proteins that are crucial factors for osteoclast performance. The network analysis including all mutated genes (132) resulted in a highly complex network very difficult to interpret, hence only bone-related candidate genes were explored.

Moreover, GO biological enrichment analysis of all mutated genes present in at least 2 AFF patients (132) emphasized cytoskeletal organization, microtubule motor activity, cilium axoneme and pyrophosphatase activity. These pathways are crucial for bone remodeling [36] and osteoclast function [37] which could exacerbate the bisphosphonate action on osteoclast performance.

The main limitation of this study is its small sample size, which precludes obtaining a robust gene signature of AFF. However, all patients analyzed in the present study were chosen according to a homogenous phenotype, thereby avoiding other underlying bone-related disorders. Further large-scale studies using NGS would be necessary to elucidate enriched pathways in the AFF propensity. Moreover, we explored coding regions since we hypothesized that rare variants altering the protein performance could be involved. Nonetheless, we cannot rule out that rare or common variants in regulatory regions and/or affecting the transcription or translation levels can participate in the AFF genetic background.

## 5. Conclusions

The main conclusion of this study is that AFF may present a multigenic background, specific to each patient, in which an accumulation of susceptibility variants may lead to a predisposition to BP-related AFF. Our analysis suggested that Wnt signaling may play a relevant role in the BP-related AFFs as half of the patients had mutations in a gene of this pathway. In silico analysis suggested a complex interaction network among the different mutated genes as well as a biological enrichment for cytoskeleton and cilium organization. WES analysis provided evidence to support the hypothesis that several genes and their interactions may be involved in the development of AFF, and, along with BP treatment and, in some cases, glucocorticoids, they may trigger the perfect storm.

## Figures and Tables

**Table 1:** Patient characteristics.

	With AFF *N* = 12	No AFF *N* = 4	*p*-Value
Mean age (years ± SD)	74.5 ± 6.1	79 ± 7.2	NS
BMI ± SD	29.7 ± 4.6	25.3 ± 3.3	NS
Time on BP (Years ± SD)	9.1 ± 4.4	9 ± 2.7	NS
Denosumab (*n*)	2	1	NS
Corticosteroid treatment (*n*)	6	0	<0.05
AFF Bilateral (*n*)	3	-	-
Previous OP fractures (*n*)	8	2	NS

Abbreviations: SD, standard deviation; BMI, body mass index; OP, osteoporosis; BP, bisphosphonate; AFF, atypical femoral fracture; NS, Non-significant.

**Table 2:** Genes with at least two individuals carrying a rare variant; Variants were prioritized based on functional prediction (excluding variants with CADD score <20, and those considered tolerated or benign by SIFT or PolyPhen_humDiv, respectively).

Genes with Rare Variants in Two AFF Cases				Genes with Rare Variants in More Than two AFF Cases	
Two Different Variants			One Variant		
Gene Name	Gene Name	Gene Name	Gene Name	Gene Name	Number of Variants and (Carriers)
*AASS*	*DNAH10*	*PSD3*	*ACADL*	*C8orf46*	1 (3)
*ABCA10*	*DNAH12*	*PTH1R*	*C1orf87*	*CHRNG*	3 (3)
*ABCA4*	*DNAH6*	*PYHIN1*	*CD1A*	*DAAM2*	3 (3, one homoz)
*ABL2*	*DYSF*	*R3HDML*	*CITED4*	*DNAH14*	4 (4)
*ADAMTS12*	*EFHB*	*RET*	*GBA*	*DNAH2*	3 (3)
*ANAPC11*	*EP400*	*RMDN1*	*IQSEC3*	*DNAH9*	3 (3)
*ANK3*	*ERCC5*	*RNF157*	*NSMAF*	*FSIP2*	3 (3)
*ANKRD40*	*FAT4*	*RNF34*	*PPP2R1B*	*HLA-DRB1*	2 (4)
*ARHGEF18*	*FBLN7*	*RTEL1*	*SERPINB2*	*HRASLS*	1 (3)
*ARID1B*	*FLJ00418*	*SCN9A*	*SPTBN1*	*IGFLR1*	2 (2, one homoz)
*ASH1L*	*GBP3*	*5-Sep*	*SYDE1*	*KRT10*	1 (5)
*ATAD2*	*GPX4*	*SH3BP2*	*TNFRSF25*	*LAMA1*	3 (3)
*ATP10B*	*HK3*	*SHROOM4*	*TRAPPC2L*	*LRP5*	4 (3)
*BIN1*	*HPS6*	*SIRT5*	*TRIM32*	*MRPS12*	1 (3)
*C10orf54*	*IGFN1*	*SLC26A9*		*NEB*	4 (4)
*C12orf42*	*IGSF10*	*SLC2A7*		*OBSCN*	5 (5)
*C14orf159*	*IGSF22*	*SLC34A3*		*TCOF1*	3 (4)
*C17orf107*	*KLHL33*	*SLC52A2*		*TNXB*	3 (3)
*C6*	*LLGL1*	*SPTBN5*		*TTN*	8 (8)
*C9orf84*	*MEX3D*	*SRCAP*		*UTRN*	3 (3)
*CA9*	*MKS1*	*TAF15*		*VEGFB*	1 (3)
*CDC42BPG*	*MMP20*	*TENM4*		*ZC3H3*	3 (3)
*CERKL*	*MSLNL*	*TJP3*			
*CHAMP1*	*NOD2*	*TMEM143*			
*CLCN2*	*NUP153*	*TNRC6B*			
*CRYBA1*	*OPLAH*	*TOPORS*			
*CTSE*	*PACSIN2*	*TSFM*			
*CUL7*	*PARD6B*	*TTC14*			
*CYYR1*	*PCDHAC1*	*ZNF34*			
*DAB2IP*	*PDE4DIP*	*ZNF646*			
*DAW1*	*PISD*	*ZNF729*			
*DHX34*	*PLA2G4D*	*ZSCAN32*			

**Table 3:** Genes involved in bone metabolism and/or AFF containing deleterious rare variants in at least two AFF patients of this study.

Gene ID	Number of Carriers	Function	Bone Association	Bibliography Source
*CUL7*	2	A core component of the 3 M complex required to regulate microtubule dynamics and genome integrity	Mutations in this gene produce the 3 m syndrome, which causes skeletal abnormalities	Genecards
*Daam2*	3	Involved in the canonical Wnt signaling, a pathway critical for bone formation and repair	SNPs in this gene are associated with estimated bone mineral density (eBMD). *Daam2* knockout mouse showed decreased bone strength	Musculoskeletal Knowledge Portal, Morris et al., 2019 [24]
*DNAH10*	2	Found in cilia and flagella; ATPase activity and microtubule motor activity	SNPs in this gene are associated with waist-hip ratio and eBMD.	Musculoskeletal Knowledge Portal
*DNAH12*	2	ATPase activity and microtubule motor activity	SNPs in this gene are associated with waist-hip ratio and eBMD	Musculoskeletal Knowledge Portal
*LAMA1*	3	A major component of the basal membrane which has been implicated in a wide variety of biological processes including cell adhesion, differentiation, migration, and signaling	Binding to cells via a high affinity receptor, laminin is thought to mediate the attachment, migration and organization of cells into tissues during embryonic development by interacting with other extracellular matrix components.	Genecards
*LRP5*	4	A co-receptor with Frizzled protein family members for transducing signals by Wnt proteins	It plays a key role in skeletal homeostasis and many bone density related diseases are caused by mutations in this gene	Genecards
*MEX3D*	2	RNA binding protein, may be involved in post-transcriptional regulatory mechanisms	Found mutated in three sisters with AFF	Roca-Ayats N, et al. 2018 [12]
*PTH1R*	2	A receptor for parathyroid hormone (PTH) and for parathyroid hormone-like hormone (PTHLH).	Involved in the Hedgehog and PTH signaling pathways in bone and cartilage development	Genecards
*SLC34A3*	2	Involved in the transporting phosphate into cells via sodium cotransport in the renal brush border membrane, and contributes to the maintenance of inorganic phosphate concentration in the kidney	Mutations in this gene are associated with hereditary hypophosphatemic rickets with hypercalciuria.	Genecards
*SPTBN1*	2	Spectrin is an actin crosslinking and molecular scaffold protein that links the plasma membrane to the actin cytoskeleton, and functions in the determination of cell shape, arrangement of transmembrane proteins, and organization of organelles	SNPs in this gene are associated with eBMD and total body BMD	Musculoskeletal Knowledge Portal
*TNRC6B*	2	Involved in cellular senescence, innate or adaptive immune system, Wnt signaling, and calcium modulating pathways	SNPs in this gene are mainly associated with lean mass. One SNP was also associated with lower lumbar spine BMD and increased risk of fractures	Karasik D, et al. 2019 [27]
*TNXB*	3	A member of the tenascin family of extracellular matrix glycoproteins	Mutations in this gene are associated with the Ehlers-Danlos Syndrome	Genecards

Genecards: https://www.genecards.org/, accessed on 9 December 2021. Musculoskeletal Knowledge Portal (MSK portal): https://msk.hugeamp.org/, accessed on 9 December 2021.

## Data Availability

Datasets generated during and/or analyzed during the current study are not publicly available but are available from the corresponding author on reasonable request.

## Supplementary Materials

The following are available online at https://www.mdpi.com/article/10.3390/genes13010146/s1, Figure S1: BinGO graph of biological function enrichment analysis using Cytoscape tool. Yellow nodes indicate significant values after FDR adjustment *p* < 0.05. Genes with at least two carriers of a rare variant were considered in the analysis (132 genes), Figure S2: Network analysis of bone-related genes mutated in at least 2 AFF patients using the AFFNET tool. All possible interactions among candidate genes were displayed. The purple star indicates a candidate gene. The red border tags those gens with z-score outside of the threshold according to constraints obtained in gnomAD. Node color represents their expression depending on the expression data obtained in the GSE63009: Osteoclastic precursor cells treated or not with bisphosphonates (alendronate or risedronate) during their differentiation into mature osteoclasts, Figure S3: Network analysis of genes mutated either in AFF patients and in three sisters from a previous study^12^ using the AFFNET tool. All possible interactions among candidate genes were displayed. The purple star indicates a candidate gene. Green star indicates candidate genes without another gene interaction. The red border tags those gens with z-score outside of the threshold according to constraints obtained in gnomAD. Node color represents their expression depending on the expression data obtained in the GSE63009: Osteoclastic precursor cells treated or not with bisphosphonates (alendronate or risedronate) during their differentiation into mature osteoclasts.
