# Supplementary material for: Gene Network of Susceptibility to Atypical Femoral Fractures Related to Bisphosphonate Treatment
Source: Genes (Basel). 2022 Jan 14;13(1):146. doi: 10.3390/genes13010146 (PMC8774942; doi:10.3390/genes13010146)
Supplement: Supplementary file 1 [file genes-13-00146-s001.zip › supplementary.pdf]

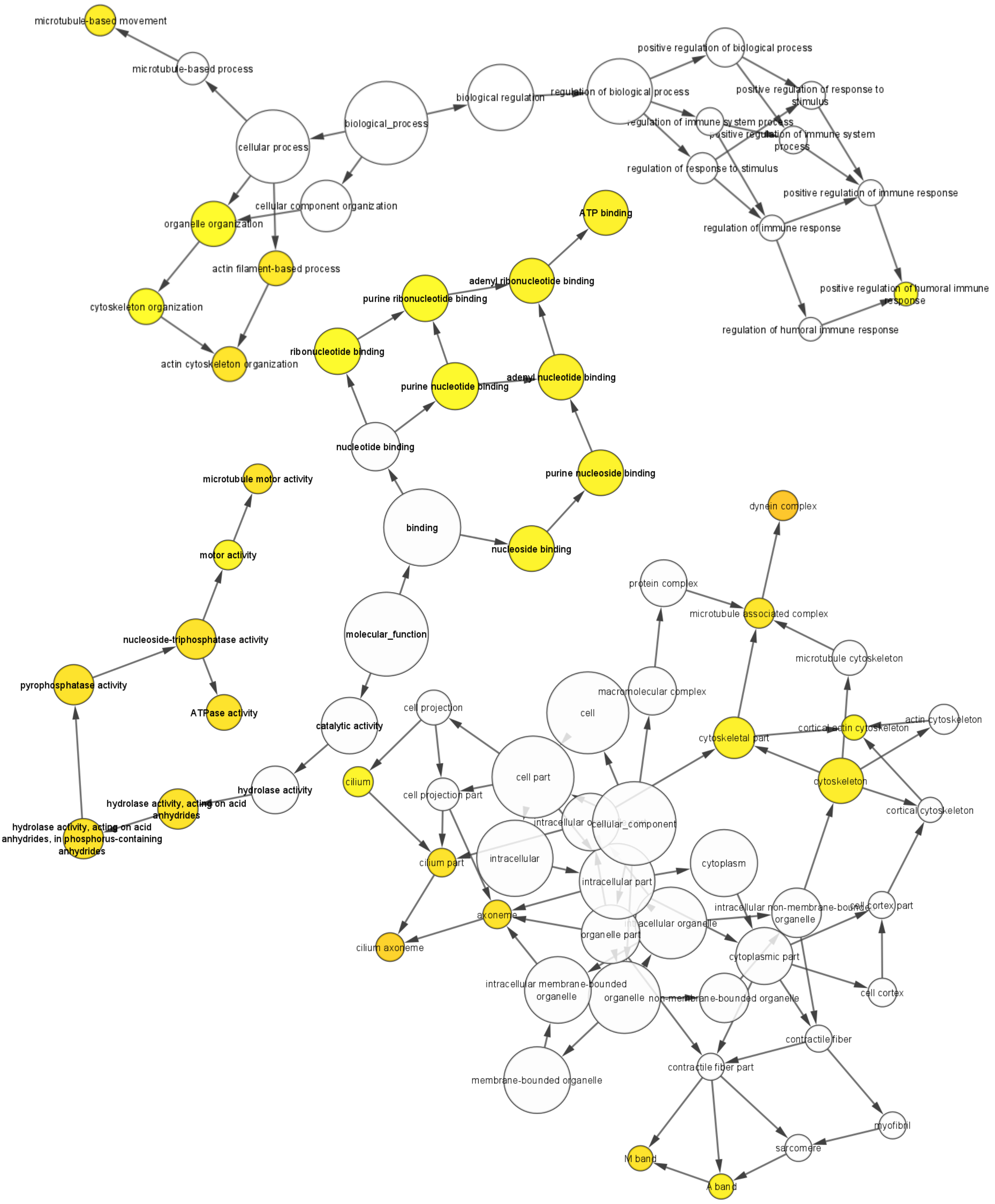

Figure S1: BinGO graph of biological function enrichment analysis using Cytoscape tool. Yellow nodes indicate significant values after FDR adjustment p<0.05. Genes with at least two carriers of a rare variant were considered in the analysis (132 genes).

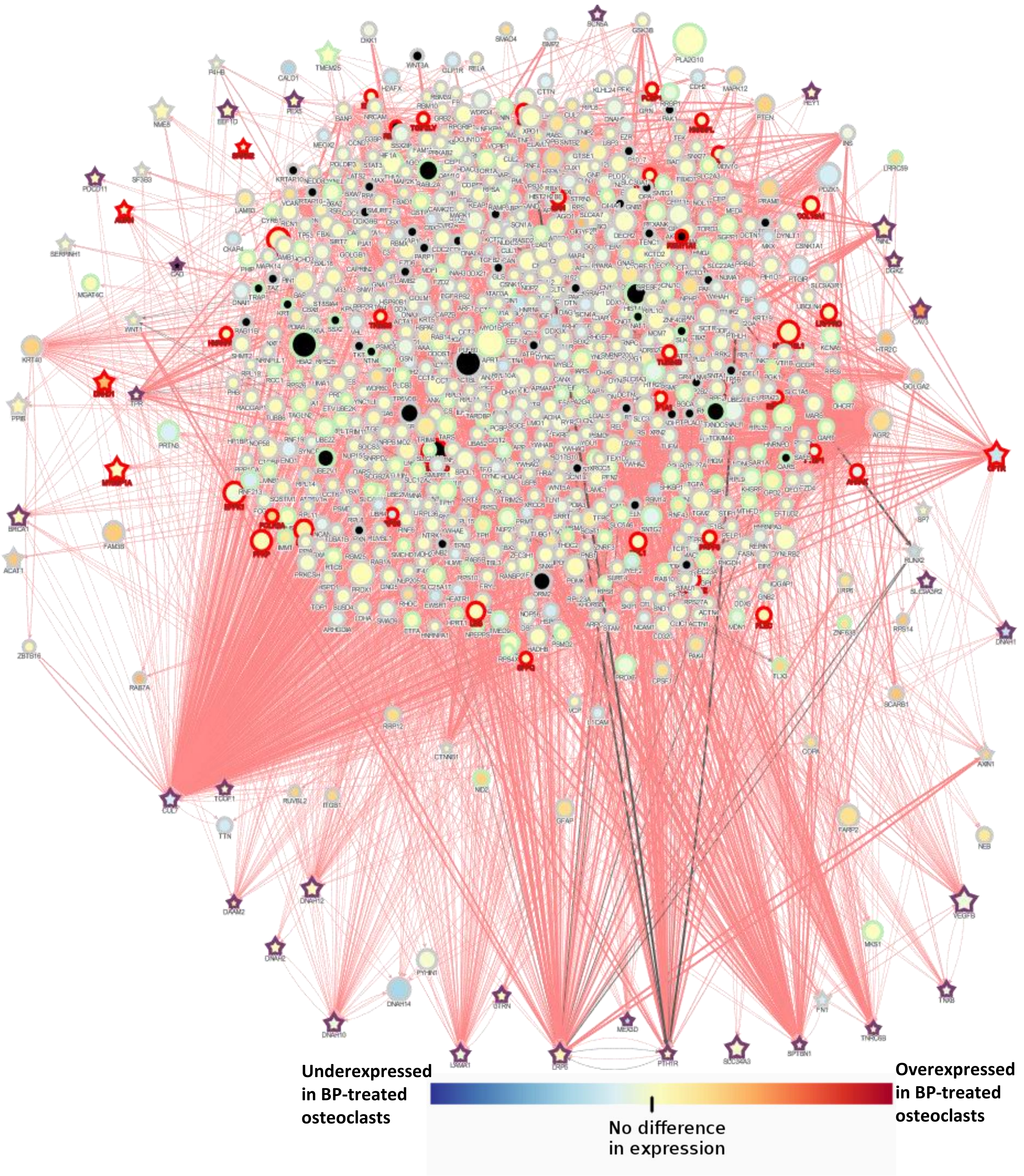

Figure S2: Network analysis of bone-related genes mutated in at least 2 AFF patients using the AFFNET tool. All possible interactions among candidate genes were displayed. The purple star indicates a candidate gene. The red border tags those gens with z-score outside of the threshold according to constraints obtained in gnomAD. Node color represents their expression depending on the expression data obtained in the GSE63009: Osteoclastic precursor cells treated or not with bisphosphonates (alendronate or risedronate) during their differentiation into mature osteoclasts.

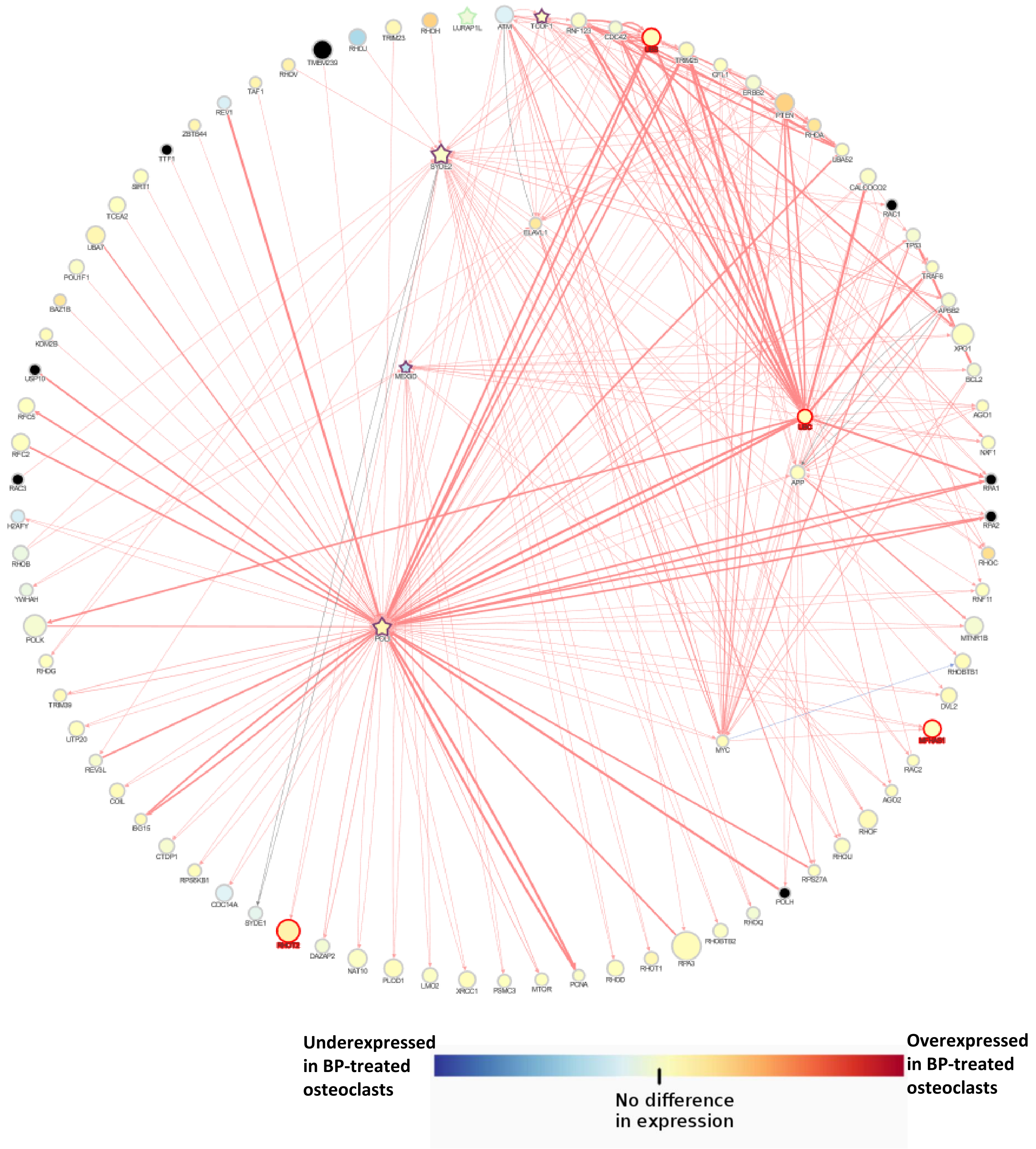

Figure S3: Network analysis of genes mutated either in AFF patients and in three sisters from a previous study[12] using the AFFNET tool. All possible interactions among candidate genes were displayed. The purple star indicates a candidate gene. Green star indicates candidate genes without other gene interaction. The red border tags those gens with z-score outside of the threshold according to constraints obtained in gnomAD. Node color represents their expression depending on the expression data obtained in the GSE63009: Osteoclastic precursor cells treated or not with bisphosphonates (alendronate or risedronate) during their differentiation into mature osteoclasts.
